# Magnetization Transfer Prepared Gradient Echo MRI for CEST Imaging

**DOI:** 10.1371/journal.pone.0112219

**Published:** 2014-11-10

**Authors:** Zhuozhi Dai, Jim Ji, Gang Xiao, Gen Yan, Shengkai Li, Guishan Zhang, Yan Lin, Zhiwei Shen, Renhua Wu

**Affiliations:** 1 Department of Medical Imaging, The Second Affiliated Hospital, Medical College of Shantou University, Shantou, 515000 Guangdong, PR China; 2 Department of Biomedical Engineering, Faculty of Medicine, University of Alberta, Edmonton, T6G 2V2, Canada; 3 Department of Electrical and Computer Engineering, Texas A&M University, College Station, TX, United States of America; 4 Department of Math and Information Technology, Hanshan Normal University, Chaozhou, 521000, Guangdong, PR China; 5 Provincial Key Laboratory of Medical Molecular Imaging, Shantou 515000, Guangdong, PR China; National Institute of Radiological Sciences, Japan

## Abstract

Chemical exchange saturation transfer (CEST) is an emerging MRI contrast mechanism that is capable of noninvasively imaging dilute CEST agents and local properties such as pH and temperature, augmenting the routine MRI methods. However, the routine CEST MRI includes a long RF saturation pulse followed by fast image readout, which is associated with high specific absorption rate and limited spatial resolution. In addition, echo planar imaging (EPI)-based fast image readout is prone to image distortion, particularly severe at high field. To address these limitations, we evaluated magnetization transfer (MT) prepared gradient echo (GRE) MRI for CEST imaging. We proved the feasibility using numerical simulations and experiments in vitro and in vivo. Then we optimized the sequence by serially evaluating the effects of the number of saturation steps, MT saturation power (B1), GRE readout flip angle (FA), and repetition time (TR) upon the CEST MRI, and further demonstrated the endogenous amide proton CEST imaging in rats brains (n = 5) that underwent permanent middle cerebral artery occlusion. The CEST images can identify ischemic lesions in the first 3 hours after occlusion. In summary, our study demonstrated that the readily available MT-prepared GRE MRI, if optimized, is CEST-sensitive and remains promising for translational CEST imaging.

## Introduction

Chemical exchange saturation transfer (CEST) imaging is a variant of magnetization transfer (MT) MRI contrast mechanism that probes the chemical exchange between dilute labile protons and bulk water [1]. Specifically, a frequency selective off-resonance radio frequency (RF) irradiation pulse is applied to preferentially saturate the exchangeable protons, and the bulk water signal is attenuated due to its exchange with saturated labile protons. Because CEST imaging is sensitive to dilute CEST agents, local pH and temperature, it can be applied in many different aspects [2]. Indeed, CEST MRI has been shown capable of detecting a host of biomolecules including glutamate [3], glycogen [4], glucose [5], glycosaminoglycan [6], esterase enzymes [7] and gene expression [8] as well as pH and temperature [9], [10], [11], [12], [13], [14], complementing the conventional MRI.

For dilute CEST agents undergoing slow and intermediate chemical exchange, the magnitude of CEST effect is typically a few percent and therefore, it is important to improve its sensitivity by optimizing the acquisition and post-processing strategies [15], [16], [17], [18], [19]. The conventional CEST MRI sequence includes a long irradiation pulse (e.g. continuous wave or RF pulse train) followed by fast image acquisition such as echo planar imaging (EPI), which is prone to image distortion, particularly severe at high field strength. In addition, CEST MRI using rapid acquisition with relaxation enhancement (RARE), fast low angle shot (FLASH) and steady-state free precession (FISP) readout have been proposed [20], [21], [22], [23]. Importantly, it often requires extensive sequence development and experimental optimization in order to address the stringent requirements of CEST MRI on RF duty cycle, irradiation duration and specific absorption rate (SAR).

Dixon et al. proposed interleaving short frequency-selective saturation and spatially selective excitation pulses for CEST imaging, and demonstrated that for short TR, CEST effect approaches its steady state over the saturation time [24]. But only paramagnetic CEST (PARACEST) agent was used to show the CEST effect and no in-vivo data was reported yet. However, the PARACEST is relatively straightforward for its negligence of RF spillover effect, which needs to be revised in vivo. In addition, because MT-prepared gradient echo (GRE) MRI is readily available, our study evaluated the dependence of its CEST MRI effect upon experimental parameters, including the number of saturation steps (Steps), MT saturation power (B1), GRE readout flip angle (FA) and repetition time (TR), for optimizing MT-prepared CEST MRI both in vitro and in vivo.

We also translated the optimized sequence and preliminary demonstrated endogenous amide proton transfer (APT) MRI [9], [25], a variant of CEST imaging, in permanent middle cerebral artery occlusion rat brains. To summarize, our study confirmed and optimized the readily available MT-prepared GRE MRI for in vivo CEST imaging.

## Materials and Methods

### A. Tissue-like CEST phantom

Tissue-like CEST phantoms were prepared for optimizing CEST MRI [17]. Briefly, 1% agarose was added to phosphate buffered saline (PBS) solution. The mixture was microwave heated, and immersed in a water bath at 45°C. After the temperature stabilized, the solution was transferred into centrifuge tubes. Creatine was added to reach final concentrations of 50, 100 and 200 mM, and their pH was titrated to 7.0. Tubes were inserted into a phantom holder filled with 1% agarose to minimize the susceptibility mismatch. In addition, another phantom containing 50 and 100 mM creatine in 1% agarose gel was prepared for sequence optimization (i.e. Steps, B1, FA and TR). Both phantoms were solidified under the room temperature prior to MRI.

### B. Animal Preparation

All experimental protocols were approved by the Ethics Committee of Shantou University Medical College and all experiments were performed in accordance with guidelines from the Chinese Animal Welfare Agency. Five adult male Sprague Dawley (SD) rats (weighted about 200 g) were underwent permanent middle cerebral artery occlusion (MCAO) by thread embolism. First, the rats were anesthetized with 3% pentobarbital, intraperitoneal injection, and immobilized in a standard rat table. Then, MCAO models were performed by inserting the 4-0 nylon suture into the lumen of internal carotid artery to block the origin of the middle cerebral artery. Animal core temperature, respiration and heart rates were monitored throughout the procedure. Another normal adult male SD rat was used for multi-slice CEST imaging. MCAO rats were euthanized with overdose of Phenobarbital by intravenous injection after Scanning. All efforts were made to minimize animal suffering and to reduce the number of animals used.

### C. Simulation of MT-prepared GRE CEST MRI

The time-dependent CEST effect for the proposed MT-prepared GRE CEST MRI sequence was analyzed by numerical simulation in Matlab (Mathworks, Natick MA). Specifically, we used Bloch-McConnell equations to simulate the two-pool proton exchange model. [26] We assumed representative relaxation parameters, with T1w = 1500 ms and T2w = 45 ms for bulk water, and T1s = 1000 ms and T2s = 15 ms for labile proton. A chemical offset of 1050 Hz was chosen for simulation as it represents the frequency offset of ensemble amide proton from endogenous proteins and peptides, observed at 7 T. We examined two exchange rates of 30 Hz and 500 Hz respectively, with the same concentrations and RF irradiation power of 1∶500 and 0.75 µT. We had TR = Tr+Ts+Ti and saturation time = n*TR. Where Tr is the relaxation delay time, Ts is the saturation time, Ti is the image acquisition time and n is the steps of TR. We had Tr = 10 ms, Ts = 10 ms, Ti = 1 ms. In comparison, we simulated the conventional continuous wave pre-saturation with EPI acquisition sequence, with the same physicochemical parameters.

### D. MRI Acquisitions

All MRI experiments were conducted using a horizontal bore (bore size 160 mm) Agilent 7 T animal MRI scanner (Agilent, VNMRS, USA) with a 63 mm internal diameter standard 1 H volume coil (one-channel) for RF transmission and reception. Field gradients: 400 mT/m in maximum 200A. The B_0_ field was shimmed using 3D gradient shimming that adjust high-order gradient shimming currents based on the derived B_0_ field map. The RF field and center frequency were calibrated in pre-scan. MT-prepared GRE MRI sequence was used for CEST imaging. First, the CEST imaging and Z-spectra were demonstrated by tissue-like phantoms and normal SD rat brain. Routine GRE MRI parameters were set: TR/TE = 26/2.3 ms and FA = 20°, unless specified otherwise. In addition, we had slice thickness = 2 mm, field of view (FOV) = 34×34 mm^2^, imaging matrix = 64×64, number of average (NA) = 1, dummy scan = 4, and bandwidth = 50 kHz. Total scan time was about 1.6 s for one slice.

### E. Phantom Optimization

CEST MRI was optimized as functions of the number of saturation steps (Steps), saturation power (B1), GRE readout FA and TR. Specifically, the Steps was varied from 32, 64, 128, 192, 256, and 512 to 1024 while the saturation power and duration were set to 0.59 µT and 20 ms. In addition, the MT saturation power was varied from 0.16 µT to 1.12 µT. Specifically, the MT saturation pulse was chosen as a 20 ms width gauss pulse, with flip angle varied from 50° to 350° with intervals of 50°, so their corresponding powers are from 0.16 µT to 1.12 µT with intervals of 0.16 µT (Steps = 256, FA = 15° and TR = 26 ms). Moreover, FA was varied from 5° to 60° with intervals of 5° or 10° (B1 = 0.59 µT, Steps = 256, and TR = 26 ms) and TR was varied from 26 to 210 ms in five steps (B1 = 0.59 µT, Steps = 256 and FA = 5°).

### F. In vivo Optimization and application

In order to be better applied in vivo, the same key parameters (Steps, B1, FA and TR) were also evaluated in five adult male MCAO SD rats in the first three hours after occlusion. The rat brain was immobilized in a standard rat cradle. Images were averaged 64 times to improve its signal to noise ratio caused by the use of volume coil. First, the number of saturation steps was varied from 64, 128, 256, 512, 1024 to 2048 while the saturation power and duration were set to 0.39 µT and 15 ms. In addition, the MT saturation power (gauss pulse, 15 ms pulse width) was varied from 0.39 µT to 1.57 µT with intervals of 0.19 µT with Step = 4096, TR = 21 ms, and FA = 5°. Moreover, FA was varied from 5° to 20° with intervals of 5° (B1 = 0.39 µT, Step = 4096 and TR = 21 ms) and TR was varied from 21, 30 to 40 ms (B1 = 0.39 µT, Step = 4096 and FA = 5°). Regions of interest (ROI) were manually drawn on the ischemic lesions and contralateral normal regions by three expert radiologists.

Optimized data were obtained in five MCAO SD rats brains based on the optimized parameters (saturation step = 4096, B1 = 0.39 µT, FA = 5°, TR = 21 ms). Total scan time was about 1.4 minutes for one image. Other routine MRI sequences: T2-weighted imaging (TR/TE = 2000/30 ms, NA = 8) and ADC maps (TR/TE = 2000/34 ms, NA = 8) with b-value of 1000 s/mm^2^ were acquired for reference.

### G. Data Processing

Images were processed using the VnmrJ 3.1A (Agilent, Santa Clara, USA) and Matlab (Mathworks, Natick MA). Z-spectra were calculated from the normalized images for ROI outlined in each phantom compartment. The scatters were drawn with mean signals as ordinate and RF offset as abscissas, smoothed using a spline function [27]. CEST images were calculated using the conventional MTR asymmetry analysis of three images: label (I_label_) and reference (I_ref_) images with RF saturation applied at the labile proton frequency (1.87 and 3.5 ppm for creatine and in vivo CEST MRI, respectively) and reference frequency (−1.87 and −3.5 ppm, respectively), in addition to a control scan without RF irradiation (I_0_). The MTRasym was calculated as [9]: MTRasym = (I_ref_−I_label_)/I_0._ Assessment of the contrast between the ischemic lesion and the contralateral normal region were performed via paired t-test. Values were presented as mean±SD, and p<0.05 was considered as statistically significant.

## Results

**Figure 1:**
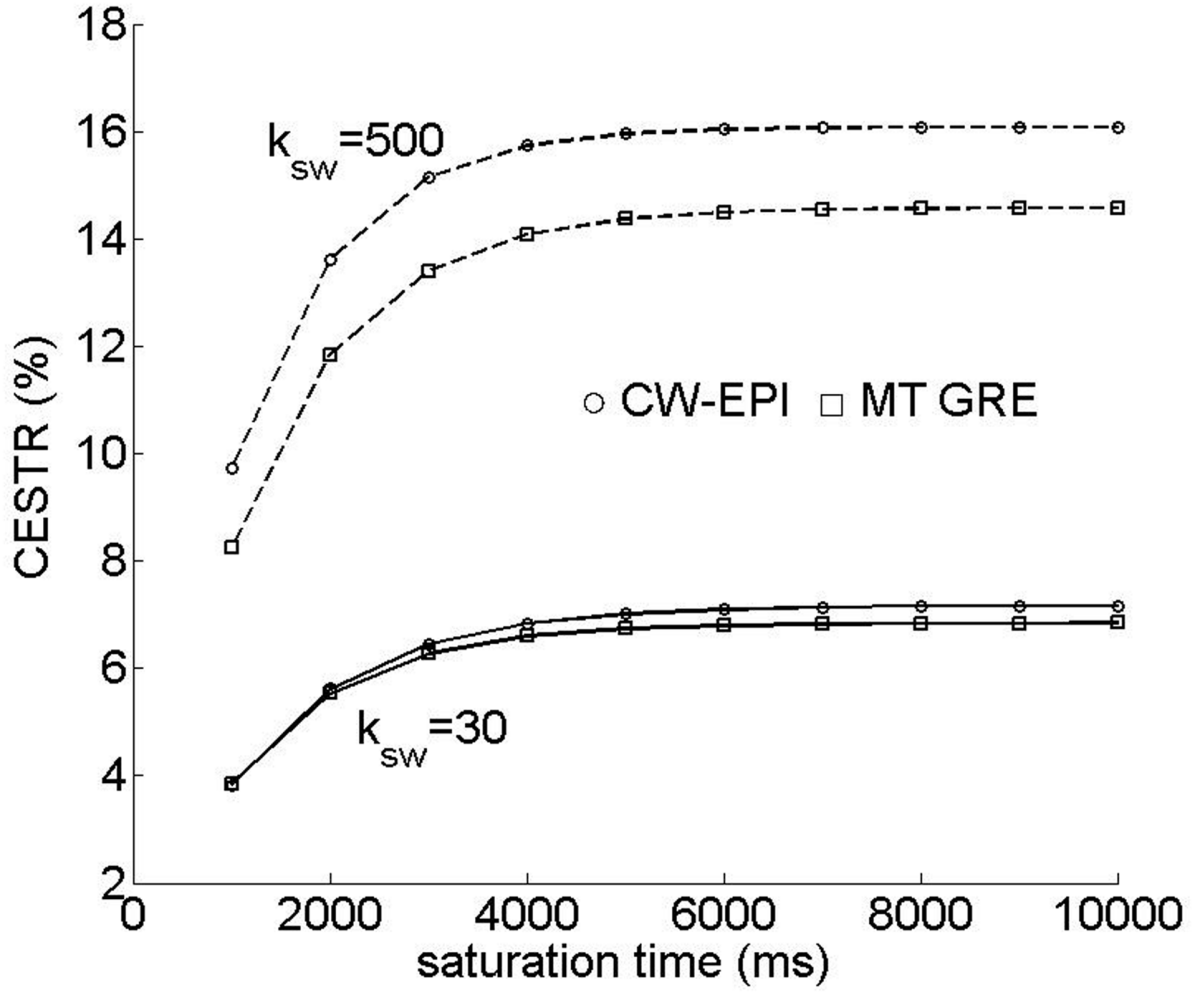
The figure shows the time-dependent CEST effect for the proposed MT-prepared GRE CEST MRI sequence and compares with that of the conventional continuous wave sequence.

Fig. 1 shows the CEST effect as a function of RF saturation time for representative exchange rates and sequences. Specifically, for short TR, the CEST effect increases during the MT duration. Although it may decrease slightly while the MT pulse is off, the CEST effect accumulates and approaches its steady state, similar to that obtainable using the conventional continuous wave saturation pulse sequence.

**Figure 2:**
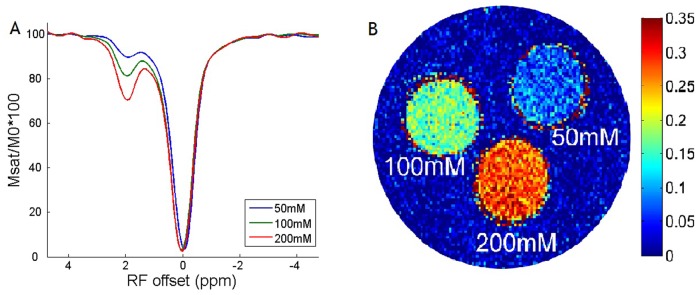
The figure shows that CEST effect can be observed at the labile amine proton chemical shift of 1.87 ppm.

Fig. 2 (A) shows Z-spectra from compartments of varied creatine concentration of the tissue-like CEST phantom. CEST-induced MRI effect can be observed at the labile amine proton chemical shift of 1.87 ppm. Fig. 2 (B) shows the CEST-weighted image of the triple-compartment CEST phantom, where the CEST effect was 7.3±3.4%, 16.7±3.2%, and 28.4±3.2% for 50, 100 and 200 mM creatine-gel compartment, respectively.

**Figure 3:**
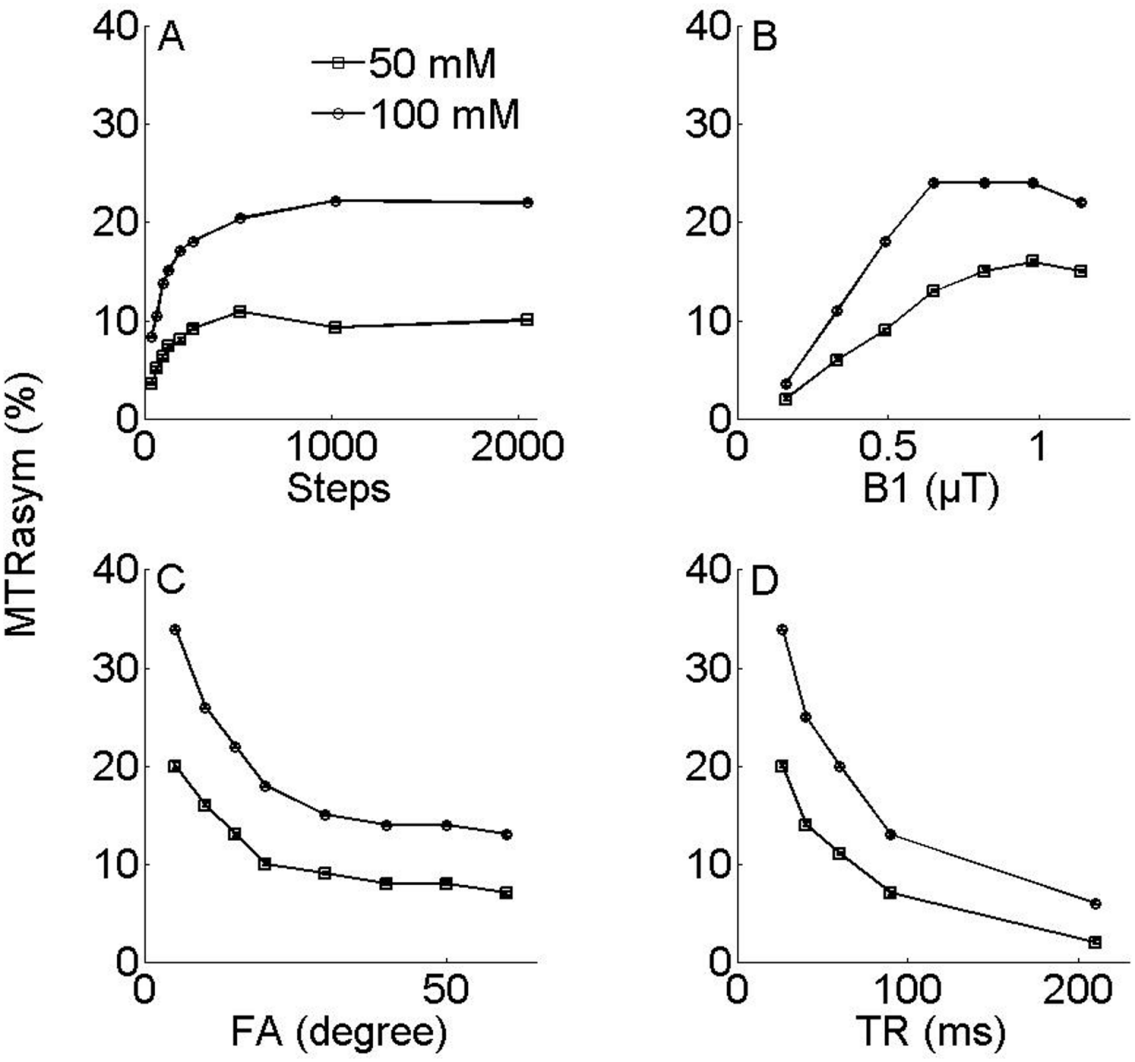
The figure shows the phantom optimizations. CEST effect approached its steady state exponentially with Steps (Fig. 3A). Whereas the CEST effect (Fig. 3B) increased with B1 and peaked at about 0.65–0.98 µT. The CEST effect decreased monotonically with FA (Fig. 3C). In addition, CEST effect decreased with TR (Fig. 3D).

Fig. 3 shows the phantom optimizations. We evaluate the CEST effect as functions of Steps, B1, FA and TR in phantoms. Specifically, CEST effect approached its steady state exponentially with Steps (Fig. 3A), indicating that CEST effect accumulated as a function of saturation step duration, that was consistent with the simulation result. Fig. 3B shows CEST effects as a function of MT saturation power increasing with B1 up to a peak at about 0.65–0.98 µT. Fig. 3C–D evaluates FA and TR dependence of CEST imaging. CEST effect decreased monotonically with FA (Fig. 3C). In addition, CEST effect decreased with TR (Fig. 3D), suggesting less effective accumulation of CEST effect at long TR due to competitive T1 relaxation. The data suggest that for the MT-prepared GRE CEST sequence, it is necessary to use small FA and short TR in order to measure CEST effect with adequate sensitivity. We propose using 5° and minimum TR at 7 T.

**Figure 4:**
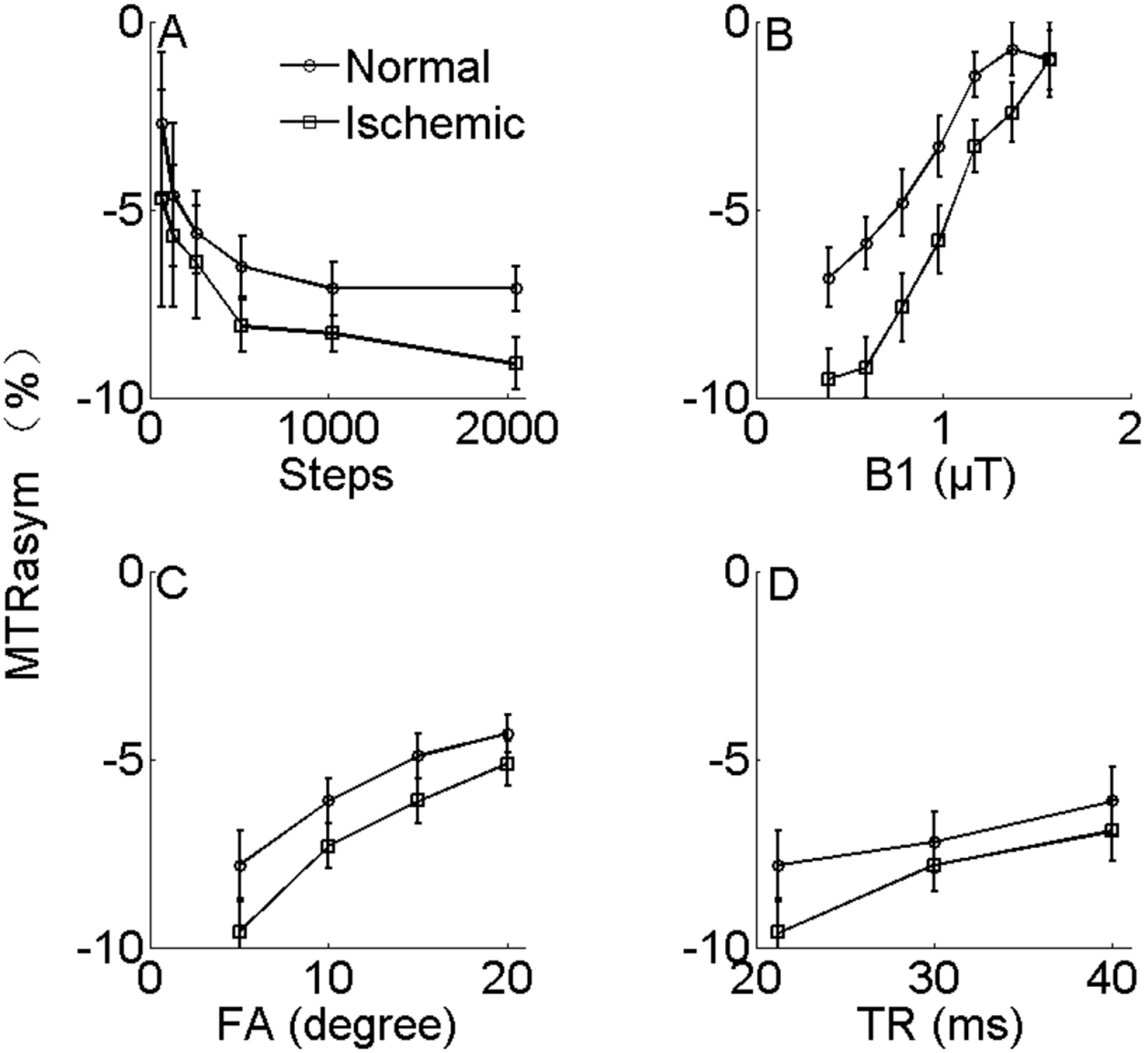
The figure shows the in vivo optimizations in MCAO SD rat brain in the first three hours after occlusion. Fig. 4A indicates that CEST effect approached its steady state exponentially with Steps in both normal and ischemic regions. Fig. 4B shows CEST effect as functions of MT saturation power (B1). The contrasts were decreasing both with increasing FA (Fig. 4C) and TR (Fig. 4D).

Fig. 4 shows the in vivo optimizations in MCAO SD rat brain in the first three hours after occlusion. The same four key parameters, including Seps, B1, FA and TR were evaluated. Unlike the results of the phantom experiments, The CEST effects in vivo were negative owning to interference with nuclear overhauser effects (NOE). We could not distinguish them in the CEST imaging with the existing technology, so we used the MTRasym contrast between ischemic lesion and normal region as the assessment index for optimization. Specifically, Fig. 4A indicates that CEST effect approached its steady state exponentially with Steps in both normal and ischemic regions. Fig. 4B shows CEST effect as functions of B1. The CEST effect increased with B1, whereas the contrasts between the normal and ischemic regions were getting smaller. The contrasts peaked at about 0.39∼0.78 µT and disappeared at 1.57 µT. Fig. 4C–D evaluates FA and TR dependence of CEST imaging. The contrasts between the normal and ischemic regions were decreased both with FA (Fig. 4C) and TR (Fig. 4D). The data also suggest that for the MT-prepared GRE CEST sequence, the optimized parameters in vivo for MCAO are as following: Steps ≥2000, B1 = 0.39∼0.78 µT, FA = 5°, TR = 21 ms.

**Figure 5:**
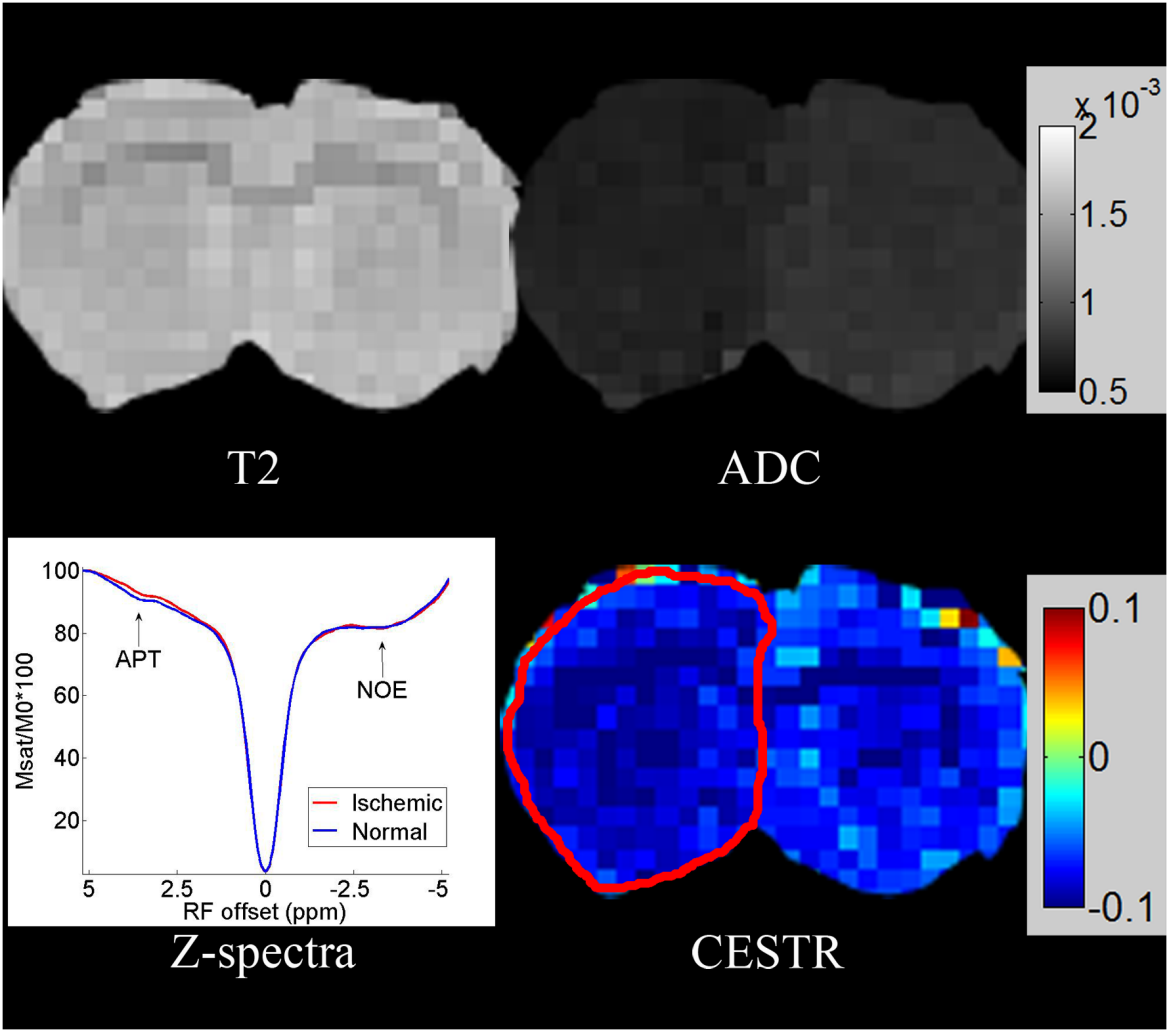
The figure shows the CEST image and Z-spectra of one MCAO SD rat brain using the optimized MT-prepared GRE MRI sequence, and compares them with other routine images. The CEST effect was verified by the Z-spectra (lower left corner). The CEST effect was reduced due to the decrease of pH in the ischemic lesion, whereas the NOE was almost the same as in the normal region.

Fig. 5 shows the CEST imaging and Z-spectra of MCAO SD rat brains (n = 5) using optimized MT-prepared GRE MRI sequence, and compare them with other routine imaging. The ischemic region was not seen in the normal T2-weighted images in the first 3 hours after occlusion. However, it could be identified in CEST images and ADC images. The CEST effect calculated from the asymmetry analysis was −9.6%±0.9% and −7.0%±0.8% for the ischemic and the contralateral normal region (p<0.05), which was verified by the Z-spectra (lower left corner). Amide proton shows a relatively clear CEST effect at about 3.5 ppm in Z-spectra. Moreover, the CEST effect was reduced due to the decrease of pH in the ischemic lesion, which was the mechanism of the contrast between the ischemic and normal region. Note that because of the interference of the NOE, there was a signal decrease on the negative axis, whereas the NOE was almost identical between the ischemic and normal region.

## Discussion

Our study found that although each brief MT pulse generates a small increase of CEST effect, it cumulates effectively for short TR over the number of saturation steps, consistent with the conclusion of Dixon et al. [24] In addition, we assessed the CEST effect for a number of experimental parameters that should help guide the experimental optimization of CEST imaging. Because the center of the k-space determines the image intensity and signal to noise ratio (SNR), initial high k-space data could be acquired during the transient period to improve the acquisition efficiency. Besides, because GRE-based image readout at short echo time is little susceptible to image distortion, it is suitable for applications at high field strength. Moreover, the interleaved MT saturation and GRE readout spread the RF energy deposition throughout the scan when compared to the conventional CEST imaging with fast image readout, which is significant in clinical application because SAR is strictly restricted. In addition, it will be very useful for Metabolite CEST like glutamate, glycosaminoglycan etc., which requires high saturation B1 that SAR can be an issue. Furthermore, because the proposed CEST imaging scheme requires little hardware modification and sequence development, it can be implemented on routine scanners and remains promising for clinical translation [28], [29].

Our study showed that CEST effects strongly depend on TR and FA both in vitro and in vivo. Short TR reduces T1- relaxation induced loss of CEST effect for more efficient cumulative effect toward the steady state CEST MRI [30]. Because CEST effect decreases with FA, the appropriate FA appears to be 5° at 7 Tesla. In addition, we observed strong dependence of CEST effects on MT saturation power. In creatine-agarose CEST phantoms, in which the protons chemical exchange rate is about 500 Hz at pH 7, the CEST effect increases with saturation power and peaked about 0.65–0.98 µT, similar to that found with pulsed-CEST imaging [16], [31]. In vivo, the amide protons chemical exchange rate is about 30 Hz, and we found that the saturation power may range from 0.39 µT to 0.78 µT for optimal contrast between the normal and ischemic regions. In addition, the B1 sensitivity for CEST imaging is relatively small around the optimal RF amplitude [10], consistent with the study of Jokivarsi et al. [32] Moreover, the spatial resolution and sensitivity were sufficient so that the apparent CEST contrast between the ischemic region and normal region could be well delineated.

It is important to note that throughout all the experiments we used a standard one channel volume coil, which allowed us to have better B0 homogeneity than that in smaller coil. The full width at half maximum were about 15 in phantoms and 30 in rat brains. Moreover, although the direct saturation effect makes CEST imaging very sensitive to B0 inhomogeneity, it can be neglected in the APT imaging due to the small saturation B1. However, for large saturation B1, it is necessary to have B0 correction. More importantly, the signal to noise ratio can be improved significantly if the coil is upgraded, and the CEST effects can be easier to be observed. In summary, our study demonstrated that optimized MT-prepared GRE MRI is suitable for in vivo CEST imaging.

## Conclusions

Our study optimized MT-prepared GRE MRI for CEST imaging by evaluating the effects of the number of saturation steps, MT saturation power, GRE readout FA and TR in tissue-like phantoms. We have also demonstrated the endogenous amide proton CEST MRI both in healthy and in MACO SD rat brains on a 7 T scanner. In conclusion, this method can provide an alternative, straightforward, and effective way to obtain CEST images on clinic MR scanners without hardware or software modifications.
